# The Mental Health Ecosystem: Extending Symptom Networks With Risk and Protective Factors

**DOI:** 10.3389/fpsyt.2021.640658

**Published:** 2021-03-18

**Authors:** Gabriela Lunansky, Claudia D. van Borkulo, Jonas M. B. Haslbeck, Max A. van der Linden, Cristian J. Garay, Martín J. Etchevers, Denny Borsboom

**Affiliations:** ^1^Department of Psychological Methods, University of Amsterdam, Amsterdam, Netherlands; ^2^Centre for Urban Mental Health, University of Amsterdam, Amsterdam, Netherlands; ^3^Faculty of Psychology, University of Buenos Aires, Buenos Aires, Argentina

**Keywords:** dynamical systems, psychopathology, resilience, complexity, biopsychosocial

## Abstract

Inspired by modeling approaches from the ecosystems literature, in this paper, we expand the network approach to psychopathology with risk and protective factors to arrive at an integrated analysis of resilience. We take a complexity approach to investigate the multifactorial nature of resilience and present a system in which a network of interacting psychiatric symptoms is targeted by risk and protective factors. These risk and protective factors influence symptom development patterns and thereby increase or decrease the probability that the symptom network is pulled toward a healthy or disorder state. In this way, risk and protective factors influence the resilience of the network. We take a step forward in formalizing the proposed system by implementing it in a statistical model and translating different influences from risk and protective factors to specific targets on the node and edge parameters of the symptom network. To analyze the behavior of the system under different targets, we present two novel network resilience metrics: Expected Symptom Activity (ESA, which indicates how many symptoms are active or inactive) and Symptom Activity Stability (SAS, which indicates how stable the symptom activity patterns are). These metrics follow standard practices in the resilience literature, combined with ideas from ecology and physics, and characterize resilience in terms of the stability of the system's healthy state. By discussing the advantages and limitations of our proposed system and metrics, we provide concrete suggestions for the further development of a comprehensive modeling approach to study the complex relationship between risk and protective factors and resilience.

## Introduction

Understanding the causal background of psychiatric problems has been a central theme for psychiatry from its beginning as a medical discipline (1–4). For the vast majority of mental disorders, no conclusive single root causes have been found (5, 6), suggesting that psychiatric conditions may result from the interaction between many distinct factors (7). As alternatives to monocausal biological and psychogenic approaches, holistic (e.g., biopsychosocial) theories have emphasized the ontological complexity of psychiatric disorders: in this view, a psychiatric disease has been conceptualized as the outcome of a dynamic interaction between biological, psychological and social variables (8–11).

Despite their attractiveness, however, holistic ideas and concepts have often been stated in general and vague terms. Critics of holistic-dynamic approaches have, therefore, stressed the gap between recognizing the complexity of psychiatric disorders on the one hand and scientific rigor on the other [e.g., (12)]. However, there is no principled reason why holistic approaches could not be thoroughly scientific. To move toward more formalized holistic models of mental health, it has become increasingly popular to look at mental health systems using the lens of ecology (13, 14). Ecosystem research studies the interactions between organisms and their environment, and is holistic in the sense that it conceptualizes these interactions as constitutive of a single integrated system (15–17). For example, according to the ecosystem approach to human development, humans are embedded within different ecological levels (18, 19). Interactions between individuals take place within a specific environmental context and are embedded within a broader cultural and sociological level.

A variety of risk and protective factors (henceforth: RP factors) exist in each of these ecological levels. Risk factors hinder optimal coping mechanisms, increasing the probability of negative outcomes when individuals are faced with adversity, while protective factors help individuals navigate adverse life events with less damage (20). RP factors are therefore closely related to the development of *resilience*, which is defined as the ability to maintain or quickly bounce back to a healthy state despite facing adversity (21, 22). Researchers have successfully identified a host of RP factors related to resilience across various domains such as (neuro)biology, personality, socio-economic factors, and family structures [e.g., (23–25)]. For example, a frequently replicated risk factor for the development of posttraumatic stress disorder (PTSD) is childhood trauma (26, 27). On the other hand, social support is an established protective factor against the development of depression in high-risk environments (28). Various brain structures and pathways have been found to be related to resilience (29). Furthermore, severe depression has consistently been associated with dysfunctions in biological stress responses, such as irregularities in the feedback-loop of the hypothalamic–pituitary–adrenal axis [HPA axis; (30, 31)].

However, in typical schematic representations of RP factors affecting mental health and resilience, it is easy to draw causal arrows between domains, such as neurobiological variables affecting psychological variables that, in turn, affect social variables. It is, however, more difficult to specify the exact nature of those causal arrows or to analyze how the system as a whole behaves as a function of these relations. Due to the multifactorial and complex nature of mental health, few would argue that the ecosystem analogy *has* to be correct in *some* way. However, current approaches are a) insufficiently precise, as suggestive visual representations of complex systems have not yet been translated into formal models, b) not operationalized, as there exist no widely accessible tools for modeling psychological resilience, and c) silent on crucial conceptual issues, such as how psychological, biological, and social factors interact or how different time scales are related.

In the current paper, we address these issues by extending the network theory of psychopathology (32, 33) with RP factors and propose an approach to analyze the resilience of the resulting system. A recent theory by Kalisch et al. (34) proposes that resilience factors target parameters of psychopathology networks. By doing so, these resilience factors influence symptom development patterns and improve resilience. We expand this idea to include both risk and protective factors and take a step forward in formalizing the system by representing it with a statistical model. We translate various effects RP factors can have on resilience to specific targets on network parameters. To analyze the resilience of this system, we introduce two novel resilience metrics for symptom networks: Expected Symptom Activity (ESA) and Symptom Activity Stability (SAS). These metrics are developed by combining standard practices in the resilience literature with ideas from the field of ecology and physics, where resilience is defined as a healthy state that is robust in stability. In the section Theoretical Framework: RP Factors Target the Architecture of Symptom Networks, we outline the theoretical framework of the proposed system, after which we will present three studies that serve as illustrations of our system and resilience metrics (see sections Study I: Analyzing Global Effects From RP Factors on the Symptom Network, Study II: Manipulating the Target Points of the RP Factors on the Symptom Network, and Study III: Empirical Illustration of a System Including Symptoms and RP Factors). Lastly, we will discuss the limitations of our proposed system and metrics and provide concrete suggestions for future research (see the section General Discussion).

## Theoretical Framework: RP Factors Target the Architecture of Symptom Networks

The main idea behind the network approach to psychopathology is that mental disorders act as a *complex* system, where psychopathology emerges from causally interacting symptoms connected in a network (35). Symptoms are typically conceptualized as being present (possibly with some degree of severity) or absent, and accordingly modeled using an Ising model (36) or an extension thereof.

In these models, it is useful to specify two types of parameters. First, an activation parameter for every *node* (i.e., the network variables, in this case, symptoms), called the *threshold* parameter, which indicates the node's internal preference to be activated (36) or, alternatively, how much pressure is required to activate the node. For example, a node such as “suicidal ideation” will have a stronger negative threshold, meaning it is more likely to be deactivated and will require more pressure to activate, than a node such as “insomnia” which is more easily activated (33). Second, a *connectivity* parameter for every estimated *edge* (i.e., the connection between variables), which indicates the weight, type, and directionality of every edge between two nodes. Edges can be strong or weak, positive or negative, and unidirectional or bidirectional (37). The set of node and edge parameters of the network model forms the *network architecture*, which describes, for example, if there are few or many edges between symptoms and if symptoms are more or less likely to activate.

Psychological networks are dynamic models, where network architecture governs symptom activation patterns (32). Activation of one symptom can lead to activation of a strongly connected neighboring symptom. If two symptoms, e.g., “fatigue” and “depressed mood,” are connected, the theory states that activation of “fatigue” increases the probability of activating “depressed mood.” The stronger the association between two symptoms (denoted in the connectivity parameter), the higher the probability that activation of one symptom leads to activation of the other symptom (38).

If external stressors (e.g., losing one's job), are sufficient to trigger symptom activation and symptoms are strongly connected, the activation of one symptom could lead to a full activation spread where the network falls into a pattern of persisting symptom activation (38). In contrast, if symptoms are not easily activated and/or weakly connected, an external stressor might lead to the activation of one or two symptoms but will not result in a full-blown depressive episode. In this way, network architecture determines the most likely symptom activation pattern (32).

Following this line of reasoning, if (1) psychopathology develops according to the network theory of mental disorders, and (2) network architecture is of paramount importance for symptom development, the next question is *how* this network architecture arises. Which factors contribute to the development of a “healthy” or “unhealthy” network architecture, increasing or decreasing the probability that a stressful event will trigger a whole symptom activation spread?

Until now, the network theory of mental disorders has mostly focused on psychopathology and symptom networks (39). However, network theory also allows one to formalize biopsychosocial ecosystem models of mental health and resilience. Recently, an answer to how network architecture might arise has been proposed by extending symptom networks with resilience factors, which are called hybrid symptom-and-resilience-factor (HSR) networks (34). These resilience factors are represented as external, protective variables influencing symptom network architecture. In this way, resilience factors affect symptom development patterns and account for individual differences in resilience (34).

**Figure 1:**
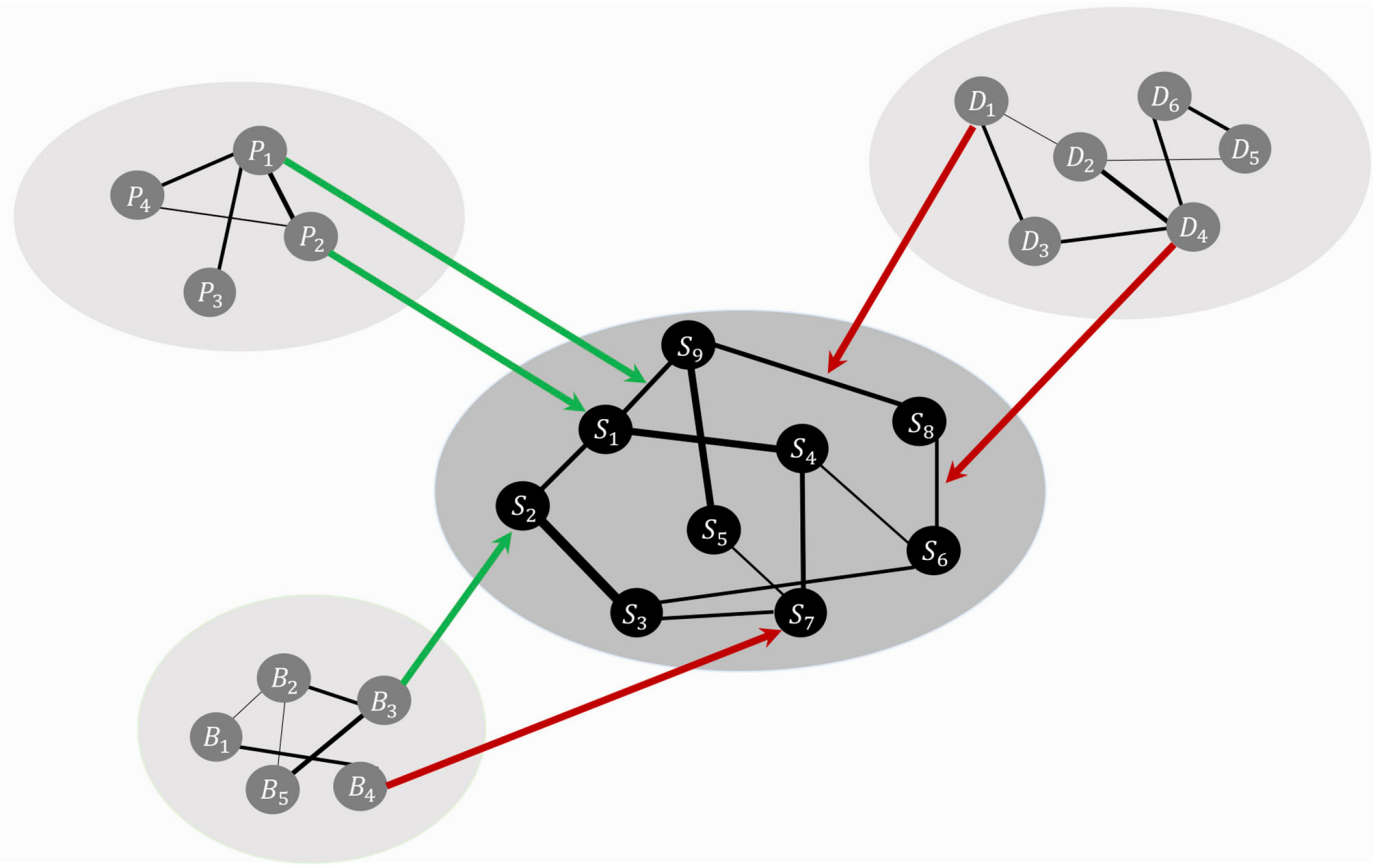
The theoretical ecosystem model of mental health. The psychopathology symptom network model, denoted with symptoms, *S*_1_ to *S*_9_, lays in the center (in black for illustrative purposes). Around the symptom network model forms a web of networks with variables from other domains, such as personality (*P*), biological (*B*), and social variables (*D*). Specific variables from other domains function as risk (red arrows) or protective (green arrows) factors, targeting node parameters or edge parameters. These risk and protective factors affect the symptom network model's architecture, thereby shaping the most likely symptom development pattern.

HSR networks need not be restricted to positive resilience factors. RP factors could both be present in these HSR networks (see Figure 1 for a representation of the theoretical model, including RP factors). For example, a protective factor such as “positive affect” could lower the strength of the connection between the symptoms “depressed mood” and “excessive worrying,” making it less likely that the activation of depressed mood will lead to the activation of excessive worrying. Contrary, vicious cognitive thought patterns (“I am worthless,” “I will never be good enough”) might affect threshold parameters of specific symptoms, making it more likely that, for example, the Generalized Anxiety Disorder (GAD) symptom “excessive worrying” will be activated. Biological factors might also influence liability for developing psychiatric disorders, and possible biological pathways have been investigated by adding genetic risk scores to psychiatric symptom networks of psychosis (40). Also, weak but differential relations have been found by adding biomarkers (estriol, cortisol, corticotropin-releasing hormone, and tumor necrosis factor alpha) to a symptom network of post-natal depression, suggesting possible symptom-specific biological pathways (41). Lastly, another example comes from the social domain, where social support has frequently been found to be a protective factor for developing Major Depressive Disorder [MDD; (42)]. The social domain variable “social support” might function as a moderator between “Depressed Mood” and “Worthlessness.” In other words, social support could lower connectivity strength between two MDD symptoms, thereby dampening the effect activation of depressed mood has on the development of feelings of worthlessness.

The theory that RP factors affect the architecture of the symptom network and, thereby, resilience (34) is a promising approach to formally study the relationship between mental health and environmental RP factors from a complex systems perspective. However, the theory has not yet been formalized or translated to a statistical model, nor has it been used to analyze empirical data. We present three studies; the first two are simulation studies, which differ in that Study I analyses the resilience of networks as a function of global effects from hypothetical RP factors (i.e., the whole network architecture is systematically altered), and Study II analyzes the resilience of networks under specific targets of hypothetical RP factors (i.e., parameters belonging to nodes with different roles in the maintenance and development of symptom activation are altered). Study III is an empirical study, in which we give an empirical illustration of the full system.

## Study I: Analyzing Global Effects From RP Factors on the Symptom Network

**Figure 2:**
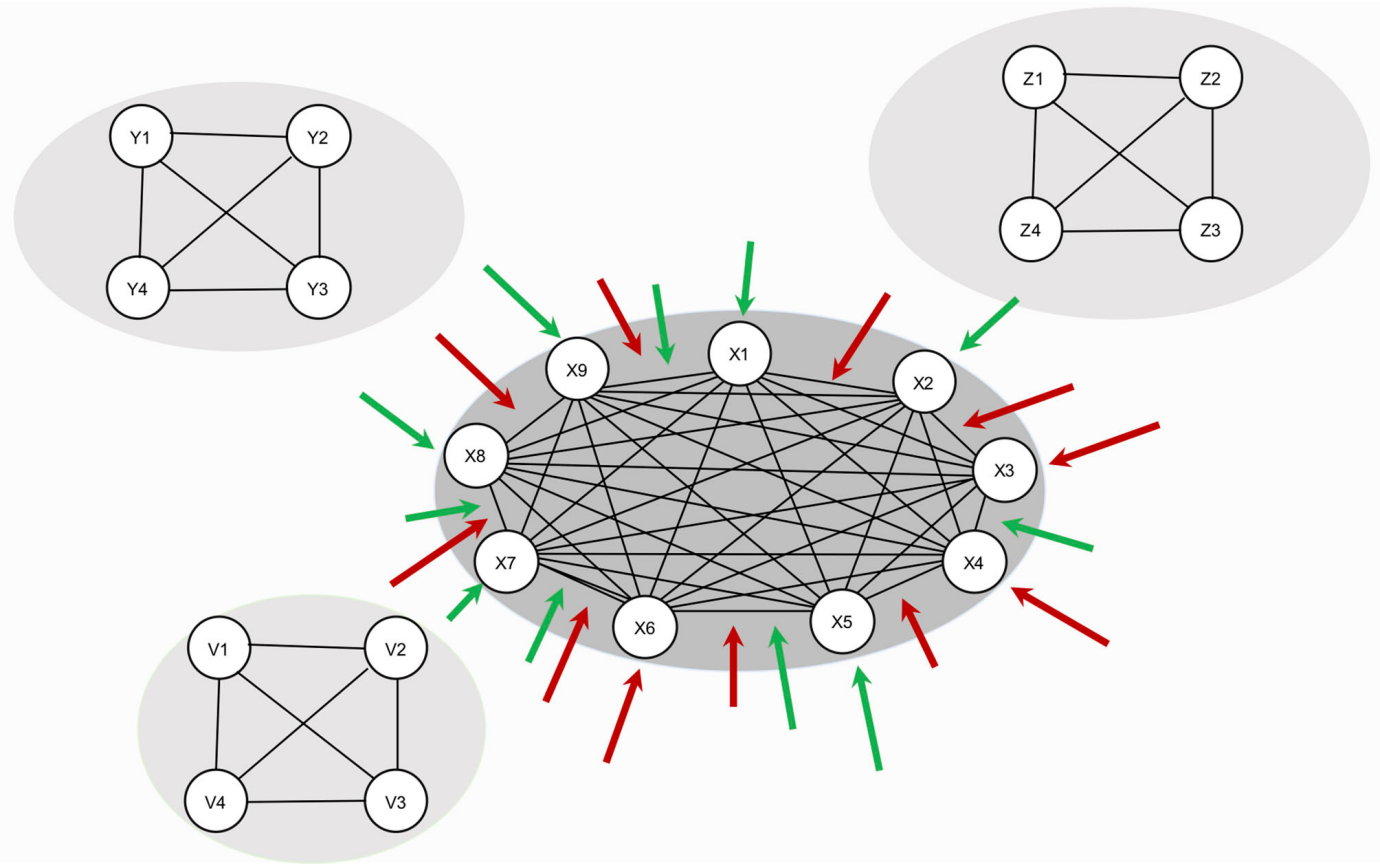
Design of study 1. The network in the center represents the symptom network (containing variables S1–S9). The three remaining networks (containing variables Y1–Y4, Z1–Z4, and V1–V4) represent hypothetical RP factors. Since no empirical data is used, all variables are denoted in statistical interpretation, without substantive labels. RP factors are assumed to cause changes in symptom network architecture, but no data on RP factors are used in the study. All RP factors are assumed to affect the symptom network architecture equally.

In this study, we investigate how the resilience of a symptom network changes under global effects of RP factors – that is, RP factors have an effect on the whole network architecture. The model in this study is illustrated in Figure 2: hypothetical RP factors (i.e., the peripheral networks containing variables Y1–Y4, Z1–Z4, and V1–V4) affect the thresholds as well as the edges of a hypothetical, fully connected psychopathology network of symptoms (i.e., the center network containing variables X1–X9 with a density of 1). Risk factors deteriorate resilience (red arrows), protective factors increase resilience (green arrows). We systematically alter the strength of the effect of RP factors and the density of the symptom network. To analyze the resilience of the symptom network model, we present two novel resilience metrics: ESA, which indicates how many symptoms are active or inactive, and SAS, which indicates how stable the symptom activity patterns are.

### Simulation Study

#### The Symptom Network Model

The symptom network model is represented by the Ising model (43). This model originates in the field of thermodynamics and ferromagnetism but has frequently been applied to represent psychological and psychiatric dynamical systems [see, for example, (36, 38, 44, 45)], due to its relative simplicity in number and type of parameters and, nonetheless, its capacity to accommodate complex phenomena. For example, in some parameter settings, the Ising model can show alternative stable states that the system converges toward, while in others, it can show linear, gradual changes (38). Other characteristics of the Ising model are that relationships are undirected (e.g., the undirected arrow between S1 and S2 implies that the relationship from S1 to S2 is equal to the relationship from S2 to S1; see Figure 2) and that all nodes of the Ising model are binary (i.e., symptoms can be inactive; denoted by a 0, or active; denoted by a 1).

A substantial advantage of the Ising model is that it is analytically solvable up to around 10 nodes (46), meaning that the full probability distribution over all states is known and that all model dynamics can be calculated from the model parameters. This allows for a complete overview of the model's behavior as a function of its architecture. For our study, this means that we know precisely how many active symptoms to expect for every parameter combination of the network, allowing us to study how the symptom network model behaves under different influences from hypothetical RP factors. We chose a network model with nine symptoms, mimicking the MDD symptoms proposed by the Diagnostic and Statistical Manual of Mental Disorders [DSM-5; (47)]. All threshold parameters have a value of −2, and all connectivity parameters have a value of 0.5.

#### Effects of RP Factors on the Symptom Network

In the proposed system, RP factors affect the resilience symptom networks by targeting edges (connectivity parameters) or nodes (threshold parameters). In our simulation, targets are operationalized by multiplying specific parameters of the symptom network with certain constants. RP factors that affect edges act as causal *moderators* (34). Such risk moderators increase connectivity parameters (i.e., multiply the edge weights with a constant >1), making it more likely that a symptom will activate its neighboring symptom. In contrast, protective moderators decrease connectivity parameters (i.e., multiply the edge weights with a constant <1).

RP factors that affect nodes act as causal *main effects*, affecting threshold parameters. Risk main effects increase a symptom's disposition for activation. Since symptom threshold parameters are generally negative, risk factors make the thresholds less negative (i.e., multiply thresholds with a constant <1). Contrary, protective main effects decrease a symptom's internal disposition for activation by increasing the negative value of threshold parameters (i.e., multiply thresholds with a constant >1).

For symmetry, the constants <1 range from 0.5 to 1 with a stepwise increase of 0.1, and constants >1 are given by the inverse of the resulting numbers. Consequently, baseline network parameters are multiplied by 11 constants: 0.50, 0.60, 0.70, 0.80, 0.90, 1, 1.11, 1.25, 1.43, 1.67, and 2. A constant of 1 represents the baseline network with no influences of risk or protective factors.

#### Network Density

**Figure 3:**
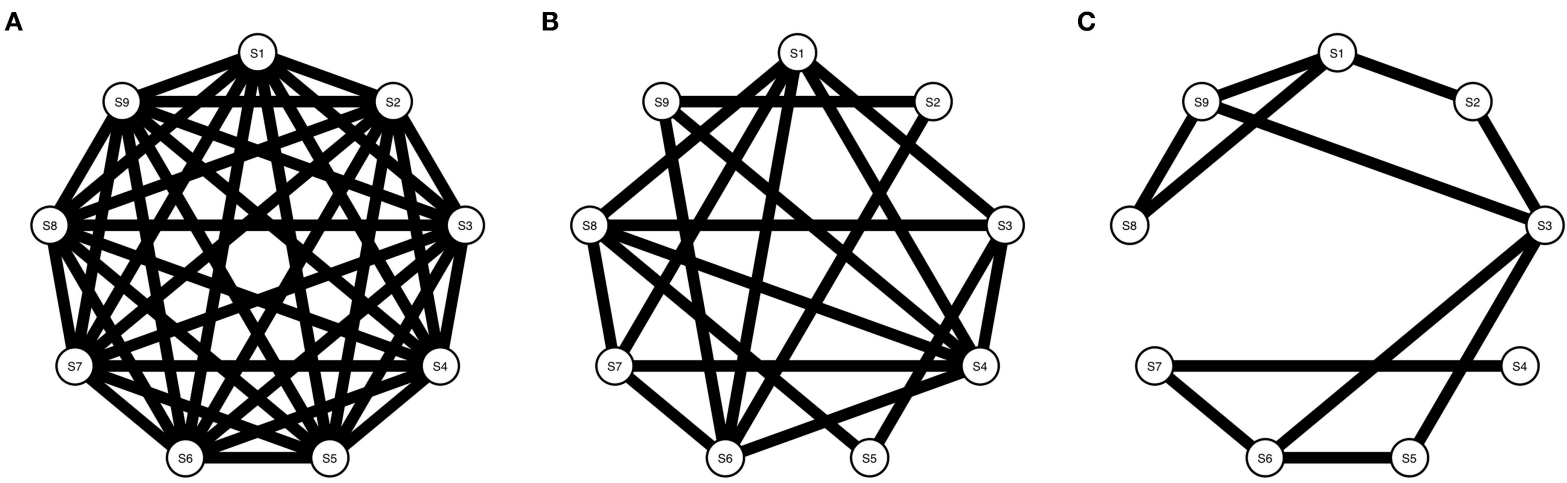
Three Ising models with varying densities. **(A)** Shows an Ising model with density = 1, **(B)** Shows an Ising model with density = 0.5, and **(C)** Shows an Ising model with density = 0.3.

Symptom activity patterns will not only depend on the strength and type of targets from RP factors on the symptom network, but also, on the density *structure* of the symptom network [i.e., the proportion of present edges relative to all possible edges; (48)]. Density influences network dynamics; the denser the network, the stronger symptoms interact and symptom activation is spread over the network (49). Therefore, we use networks with three different densities (i.e., 1, 0.5, and 0.3) in our simulations (see Figure 3).

#### Metrics to Assess Resilience

To assess the resilience of our hypothetical symptom network, we introduce two novel resilience metrics. The ESA represents the mean sum score of active symptoms as a function of the network's underlying probability distribution. This informs us whether a network is likely to be in a healthy state (i.e., a low ESA due to weak symptom activity) or an unhealthy state (i.e., a high ESA due to strong symptom activity). Symptom levels are often used to assess the validity of resilience questionnaires by relating resilience scores to the severity of mental disorders (50, 51). The rationale behind this is that individuals who score high on protective factors and/or score low on risk factors are more likely to develop fewer symptoms when faced with stressful life events than those who score low on protective factors and/or high on risk factors.

Resilience, however, is defined as the ability to maintain a healthy state (i.e., a low sum score) and quickly bounce back to a healthy state after facing adversity. In other words, a resilient system is characterized by a low ESA and *stable symptom activity*. To capture the latter characteristic, we introduce our second resilience metric, SAS, which involves the variability of the symptom activity pattern. Variability of symptom activity is an important aspect of resilience since the mean sum score can result from different activation patterns. For example, in a system with nine symptoms, a mean score of 3 could be the result of consistently moderate or highly unstable symptom activity patterns. This means that a symptom network is resilient if ESA has a low value and SAS has a high value: in that case, the dominant state of the network is one in which symptoms are stably absent^1^.

SAS is related to a model's *entropy*, which has been used as an indicator of stability in dynamic systems theory. Entropy is a measure of the probability of each possible state of the system, based on the parameters of the system (44, 52). If entropy is high, many states are equally likely, which indicates that the system's dynamics will be unstable, switching between many possible states. Contrarily, if entropy is low, only a few states have a high probability of occurring, meaning the system's behavior will be more organized and stable.

Symptom activation patterns follow from the probability distribution of the Ising model. The Ising model for two nodes (*X*_1_, *X*_2_) is given by formula (1), which extends for models with *n* nodes (53):

(1) $$\begin{array}{r} P\left(X_{1},\;X_{2}\right)=\;\frac{1}{Z}\;\exp\left\{\tau_{1}X_{1}+\tau_{2}X_{2}+W_{12}X_{1}X_{2}\right\} \end{array}$$

In this formula, *X*_1_ and *X*_2_ are elements of {0,1}, *P*(*X*_1_, *X*_2_) is the probability of the event (*X*_1_, *X*_2_), τ_1_ denotes the threshold parameter of the node *X*_1_, and *W*_12_ denotes the edge weight parameter of the neighboring nodes *X*_1_ and *X*_2_. *Z* is a normalizing constant denoting the sum of the potentials of all possible states. The probability distribution for *n* = 9 can be calculated by a generalization of formula (1).

ESA is calculated by taking the expected value E(.) of the probability distribution:

(2) $$\begin{array}{r} E\left(Y\right)=\mu =\overset{n}{\underset{i=0}{\sum }}P\left(Y_{i}\right)Y_{i} \end{array}$$

Where *Y* represents the number of active symptoms in the network (i.e., *Y* ranges over all possible sum scores; in our case from 0 to 9), *Y*_*i*_ represents a possible sum score *i*, and *P*(*Y*_*i*_) represents the corresponding probability of *Y*_*i*_ given a specific network architecture. This probability distribution is implemented in the *IsingSampler* package in the R-programming environment (46).

SAS is calculated by taking the inverse of the standard deviation σ of the expected value *E*(*Y*):

(3) $$\begin{array}{r} \sigma =\sqrt{\overset{n}{\underset{i=0}{\sum }}{\left(Y_{i}-\mu \right)}^{2}\;P\left(Y_{i}\right)} \end{array}$$

The standard deviation is a scaled variability metric. We take its inverse to align the magnitude of SAS with its interpretation: low SAS indicates weak stability, and high SAS indicates robust stability. Taking a standard deviation of 1 as a reference, SAS – the inverse of the standard deviation – is also 1. When the standard deviation is larger than 1, SAS will be <1, indicating that the stability is lower. When the standard deviation is smaller than 1, SAS will be >1, indicating that the stability is higher. We calculate *P*(*Y*_*i*_) for every change of the network architecture using *IsingSampler* (46).

ESA and SAS will be calculated for all 11 network architectures, for all three networks with different densities.

### Results

**Figure 4:**
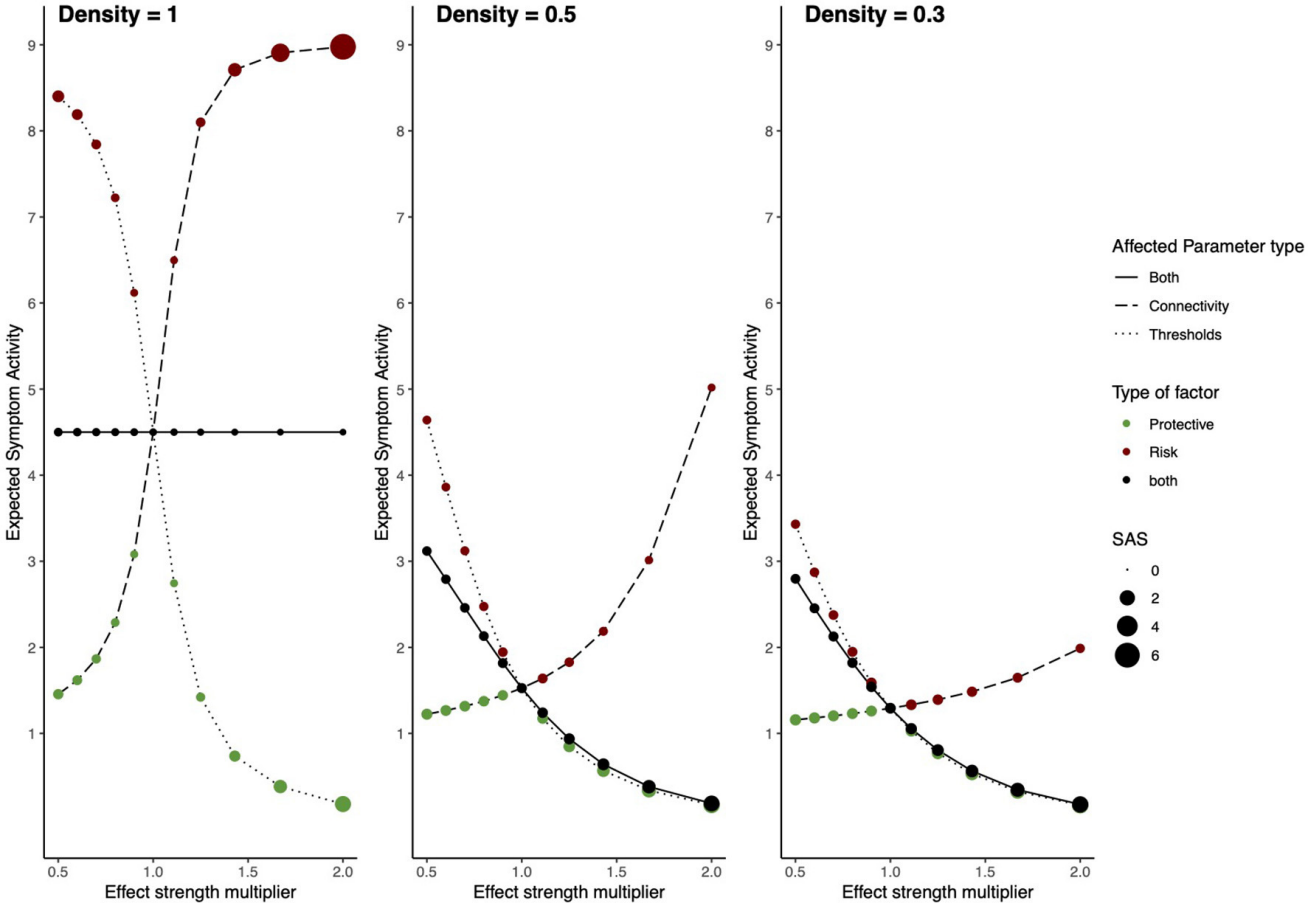
Risk and protective factors affecting Ising symptom network dynamics. The behavior of an Ising model under the influences of hypothetical RP factors for three different network densities. The left panel shows a network with density = 1, the middle panel a network with density = 0.5, and the right panel a network with density = 0.3. The x-axis denotes the value of the constant with which network architecture is multiplied. The y-axis denotes ESA. Line type represents which parameters are multiplied; threshold parameters, connectivity parameters, or both. The color of circles represents the type of hypothetical RP factor which influences network parameters: red circles represent risk factors, green circles, protective factors, and black circles represent both factors. The size of the circles represents SAS.

Results for all alterations (i.e., strength of effect of RP factors and density) on the architectures of the networks are displayed in Figure 4. Table 1 shows the results for the extremes of RP factor influences, namely when the multiplier is equal to 0.5 or 2.

**Table 1:** Network resilience for different densities and influences from risk and protective factors.

**Factor type**	**Density** **=** **1**		**Density** **=** **0.5**		**Density** **=** **0.3**		**Multiplier**
	***ESA***	***SAS***	***ESA***	***SAS***	***ESA***	***SAS***	
Baseline	4.5	0.34	1.53	0.74	1.29	0.86	1
Moderator							
Risk	8.98	6.47	5.02	0.39	1.99	0.60	2
Protective	1.46	0.77	1.22	0.91	1.15	0.96	0.5
Main Effect							
Risk	8.40	1.08	4.64	0.51	3.43	0.59	0.5
Protective	0.18	2.26	0.17	2.39	0.17	2.44	2
Both	4.5	0.49	3.12	0.62	2.80	0.67	0.5
	4.5	0.23	0.19	2.16	0.18	2.32	2

*ESA stands for Expected Symptom Activity, which describes the level of symptom activity. ESA ranges between 0 and the total number of symptoms, in this case, 9. Low ESA means a low level of activity, indicating a healthy state. SAS stands for Symptom Activity Stability, which describes the stability of symptom activation patterns. SAS is computed as the inverse standard deviation, meaning an SAS of 1 indicates a standard deviation of 1. SAS <1 indicates decreasing stability (increasing standard deviation), and SAS >1 indicates increasing stability (decreasing standard deviation). A system is resilient when ESA is low and SAS is high, as this indicates a low level of symptom activation with robust stability*.

For the model with density = 1, RP main effects and moderators strongly affect the resilience of the symptom network. In the absence of RP effects (i.e., multiplier = 1) ESA is moderate, and SAS is low, meaning that symptom activity is moderate but unstable. Protective factors decrease ESA and increase SAS, meaning that they push the network toward a resilient state. Contrary, risk factors strongly increase ESA and SAS, meaning that they push the network toward a stable state of high symptom activation. This means that as RP factors affect network parameters, symptom activity increases or decreases, and symptom development patterns become more stable. When RP factors simultaneously alter both connectivity and threshold parameters, ESA remains around its baseline value, with low ESA, indicating unstable activity patterns.

Dynamics change for the model with a density of 0.5. In the absence of RP factor effects (i.e., multiplier = 1) ESA is low, and SAS is moderate, meaning symptom activity patterns are low and relatively stable. However, risk moderators affecting edges increase ESA to moderate symptom activity and decrease SAS, meaning that risk moderators push the system toward an unhealthy and unstable state. Since there are fewer present edges that can be targeted by moderators, their effect on ESA is smaller compared to the fully connected network. This means that the network gets pushed into moderate symptom activity with corresponding instability. Main effects targeting thresholds have a more substantial effect on resilience, as they still target all threshold parameters. Protective main effects push the system in the same resilient state as the former model with density = 1.

The model with density = 0.3 follows similar dynamics as the former model with density = 0.5; however, ESA changes within a more restricted range, meaning effects from risk and protective factors on ESA are smaller.

### Discussion

In Study I, we investigated the resilience of symptom networks with varying densities and different degrees of the effect of RP moderators and main effects by inspecting ESA and SAS. Results from this simulation study show that the resilience of the network changes as a result of RP effects. However, network density also strongly affects how resilience changes. When density is 1 (i.e., a fully connected network) and risk factors target the network, ESA and SAS increase. This means that the model is in a disorder state with full symptom activity and is unlikely to recover from this. Contrary, when protective factors target the network, ESA decreases, and SAS increases. This means that the network shows strong resilience, as symptom activity is low, but stability is high.

However, as density decreases, the network's ESA also decreases, meaning that it never shows full activity in our simulations. Risk factors, especially moderators (i.e., affecting edge parameters), increase ESA and decrease SAS, implying that stability decreases as risk factors gain more influence. When both RP factors are present, the main factors affecting thresholds have a more substantial influence on ESA than moderators affecting connectivity parameters. This is due to the fact that there are fewer present edges moderators can influence, and therefore, their effect on symptom activation patterns is smaller.

## Study II: Manipulating the Target Points of the RP Factors on the Symptom Network

**Figure 5:**
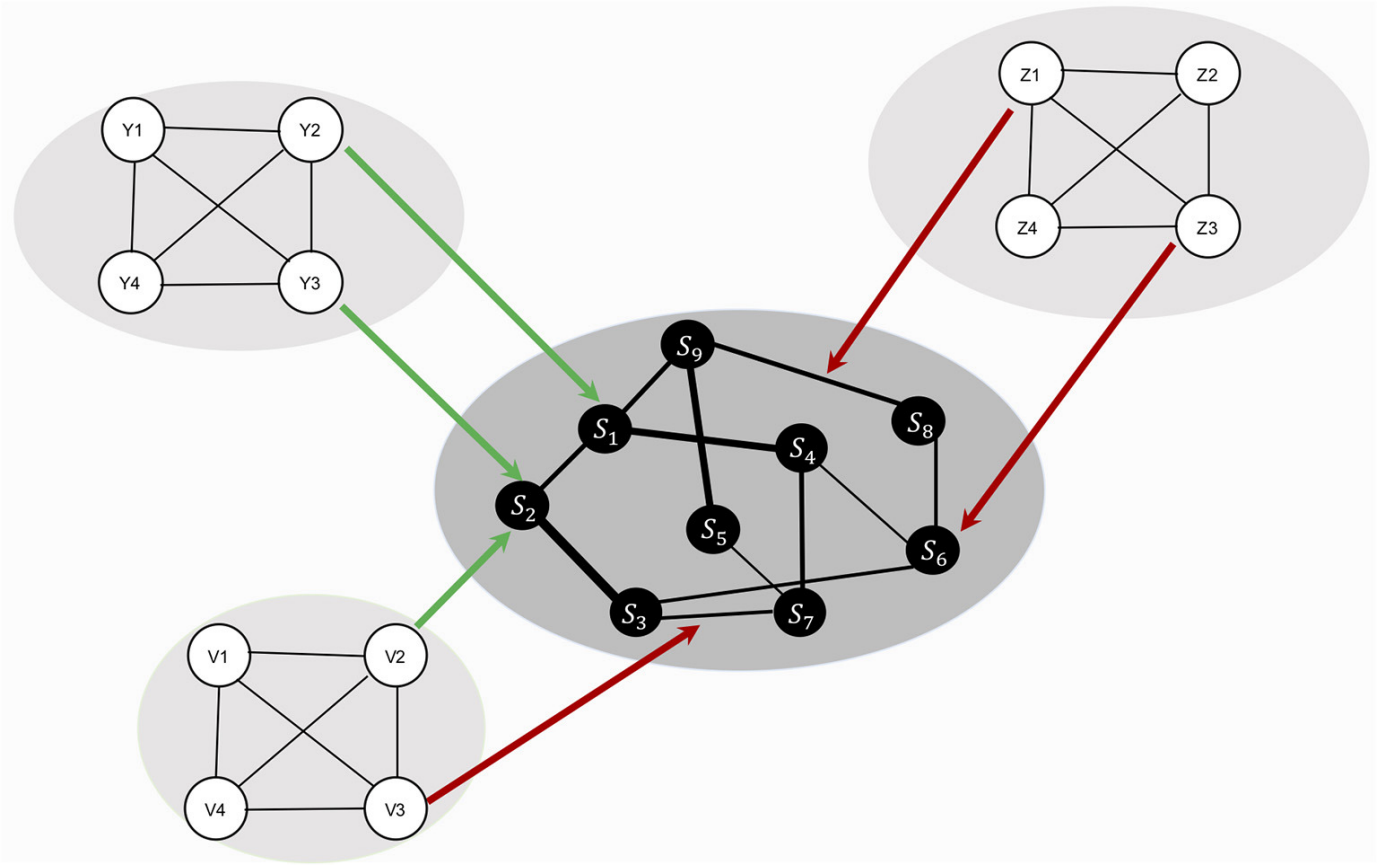
Design of study 2. The center network represents the symptom network (variables *S*_1_ to *S*_9_). The symptom network is estimated from empirical data. Therefore, edge and node parameters differ, leading to different roles symptoms have in the spread of symptom activity. The three remaining networks (containing variables Y1–Y4, Z1–Z4, and V1–V4) represent hypothetical RP factors. RP factors are assumed to change symptom network architecture, by systematically targeting symptom network parameters. We study how different target points from RP factors on symptom network architecture affect resilience, by multiplying specific symptom network parameters with constants.

A fundamental principle of network theory is that nodes differ in how important they are in maintaining and developing symptom activity (32, 54). In this study, we investigate how the resilience of a symptom network changes when target points of RP factors affect parameters belonging to nodes that have a strong or weak role in symptom activity spread. The model in this study is illustrated in Figure 5: hypothetical RP factors (i.e., the networks containing variables Y1–Y4, Z1–Z4, and V1–V4) affect specific threshold and edge parameters of the psychopathology symptom network (i.e., the center network containing variables *S*_1_ − *S*_9_). The symptom model is estimated from empirical data to obtain plausible network parameters that differ per node and edge (i.e., the symptoms vary in their importance on symptom activity spread). We systematically alter parameters belonging to nodes with a weak or strong role in the symptom network.

### Simulation Study

#### Data

Psychiatric symptoms are measured with the 27-item Symptom Checklist [SCL-27; (55)]. The SCL-27 is a multidimensional screening instrument, functioning as a validated abbreviation of the 90-Symptom Checklist (56). It consists of 27 items measuring symptoms on six dimensions: (I) depressive symptoms, (II) dysthymic symptoms, (III) vegetative symptoms, (IV) agoraphobic symptoms, (V) symptoms of social phobia, and (VI) symptoms of mistrust. Symptom descriptions can be found in Appendix 1 in the Supplementary Material. Symptoms are measured on an ordinal scale with five levels. Participants were part of an Argentinian study on mental health and were recruited via probability sampling (57). Number of participants is 1,469 (female = 875, male = 579, other = 15). The questionnaire was administered online.

#### The Symptom Network Model

**Figure 6:**
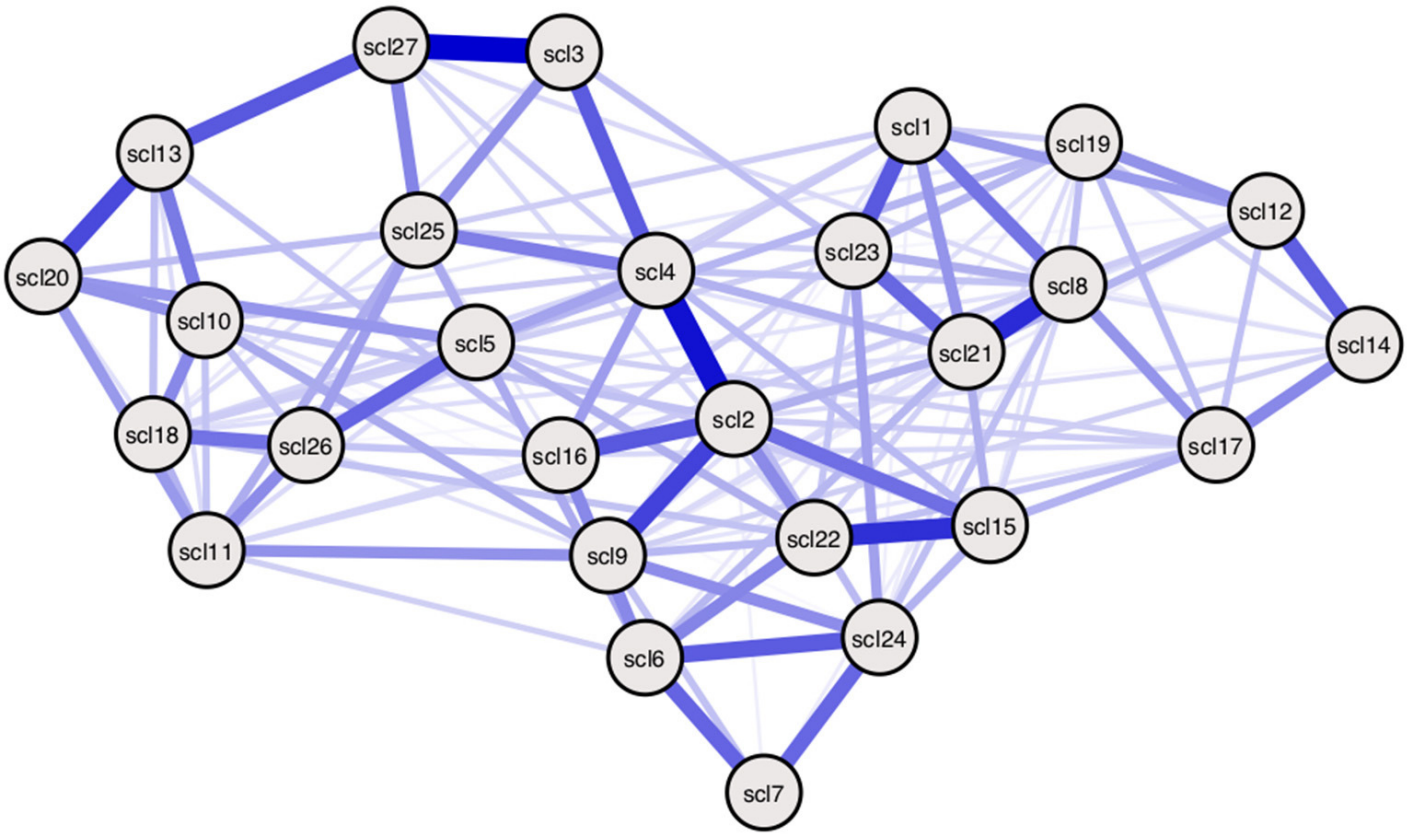
Empirically estimated SCL-27 symptom network. Empirically estimated Ising model using SCL-27 symptom data. Blue edges represent positive associations between nodes (36). The width of edges and color intensity represents the strength of edges, showing the connectivity parameters in this estimated model differ for every pair of nodes. Threshold parameters differ per node. Symptom descriptions can be found in Table 3 of Appendix 1 in the Supplementary Material.

An Ising model is used to estimate the network model (see Figure 6). In order to estimate the Ising model, the data need to be binarized. The following rule is used: responses indicating no or modest symptom presence are recoded with a 0, responses indicating moderate or high symptom presence are recoded with a 1. Thus, {0, 1, 2} --→ 0, {3, 4} --→ 1. The model is estimated using the *IsingFit* package in the R-programming environment (58).

#### Calculate ESA and SAS From Simulated Ising Model Dynamics

Since the estimated Ising model consists of 27 nodes, the underlying probability distribution cannot be calculated analytically. Instead, we need to simulate data points using a sampling method. The *IsingSampler* package in the R-programming environment includes three sampling methods to simulate states from an Ising model. We will use the Metropolis-Hastings algorithm (59). The chain starts with random values for every node, consisting of a 0 or a 1 (indicating presence/absence of the symptom). Then, for every iteration, a node is set to its opposite response option, and the probability of that node being in the opposite option given all other node values and parameters is calculated. In this way, the chain converges to the most probable state of the model based on its parameters. We use 1,000 iterations for every chain.

ESA is calculated by taking the mean sum score and SAS by taking the inverse standard deviation of the 1,000 simulated data points.

#### Strong Nodes and Weak Nodes Condition

Some nodes could be more involved than others in the spread of symptom activity when they are more central than others (33, 60) Centrality indices describe how strong nodes are connected with other nodes and/or how many connections they have with neighboring nodes (61). Nodes with many strong associations are hypothesized to have a more substantial influence on symptom development patterns. Different centrality indices exist, but, currently, *node strength* is the most stable one (61). Therefore, we use node strength to determine which nodes are targeted by RP factors. Node strength centrality is calculated by taking the sum of all absolute edge weights a node is directly connected to (62).

**Figure 7:**
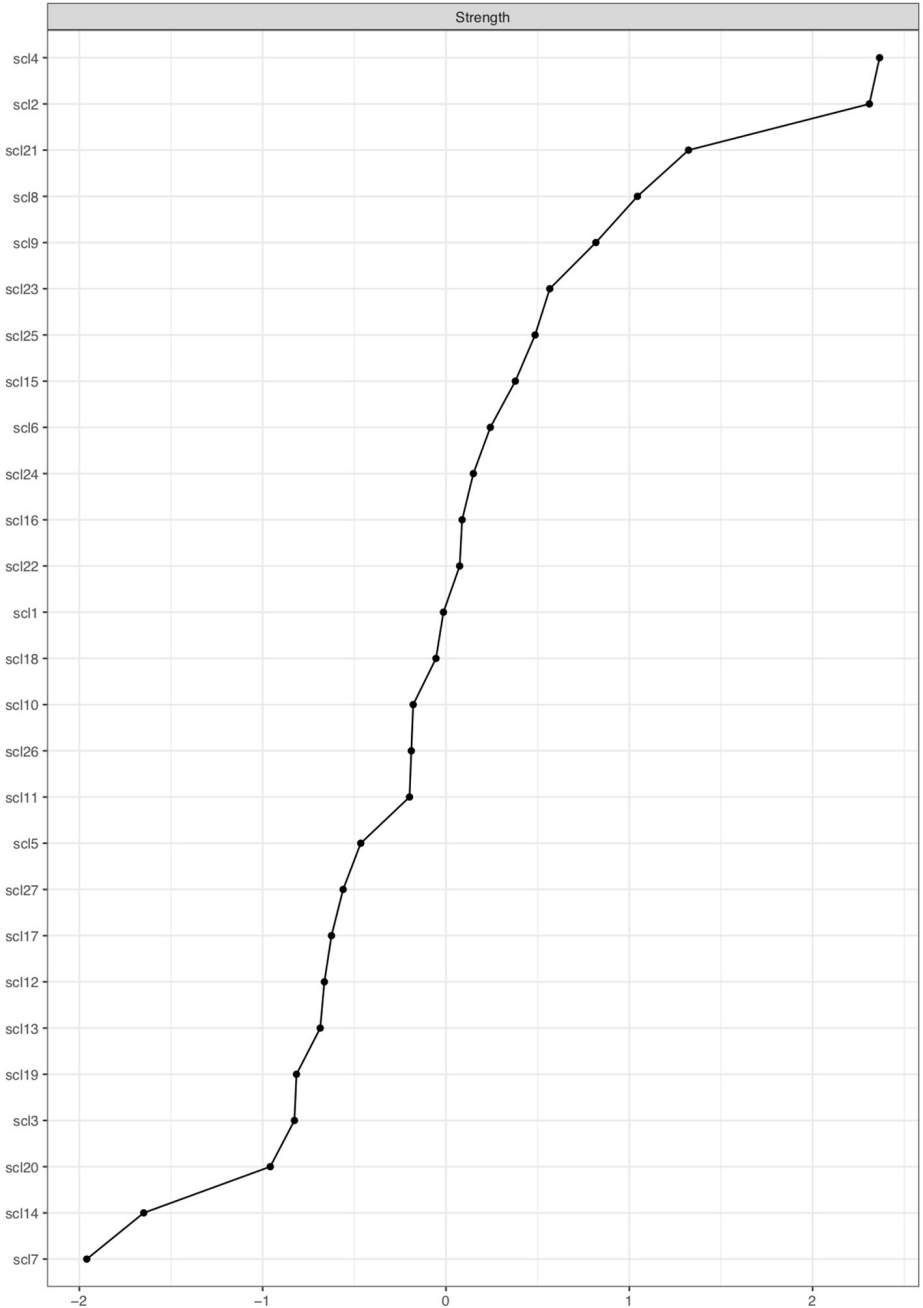
Centrality plot showing node strength of SCL-27 symptoms. The x-axis shows node strength on standardized z-scores; the y-axis shows all SCL-27 variables. The variables are ranked from highest to lowest node strength.

Figure 7 shows the node strength indicator for every node, ordered from high node strength to low node strength. The five nodes with the highest node strength are SCL-2, SCL-4, SCL-8, SCL-9, and SCL-21, which will be called *strong nodes*. The *weak nodes* are the five nodes with the lowest node strength: SCL-3, SCL-7, SCL-14, SCL-19, and SCL-20.

**Figure 8:**
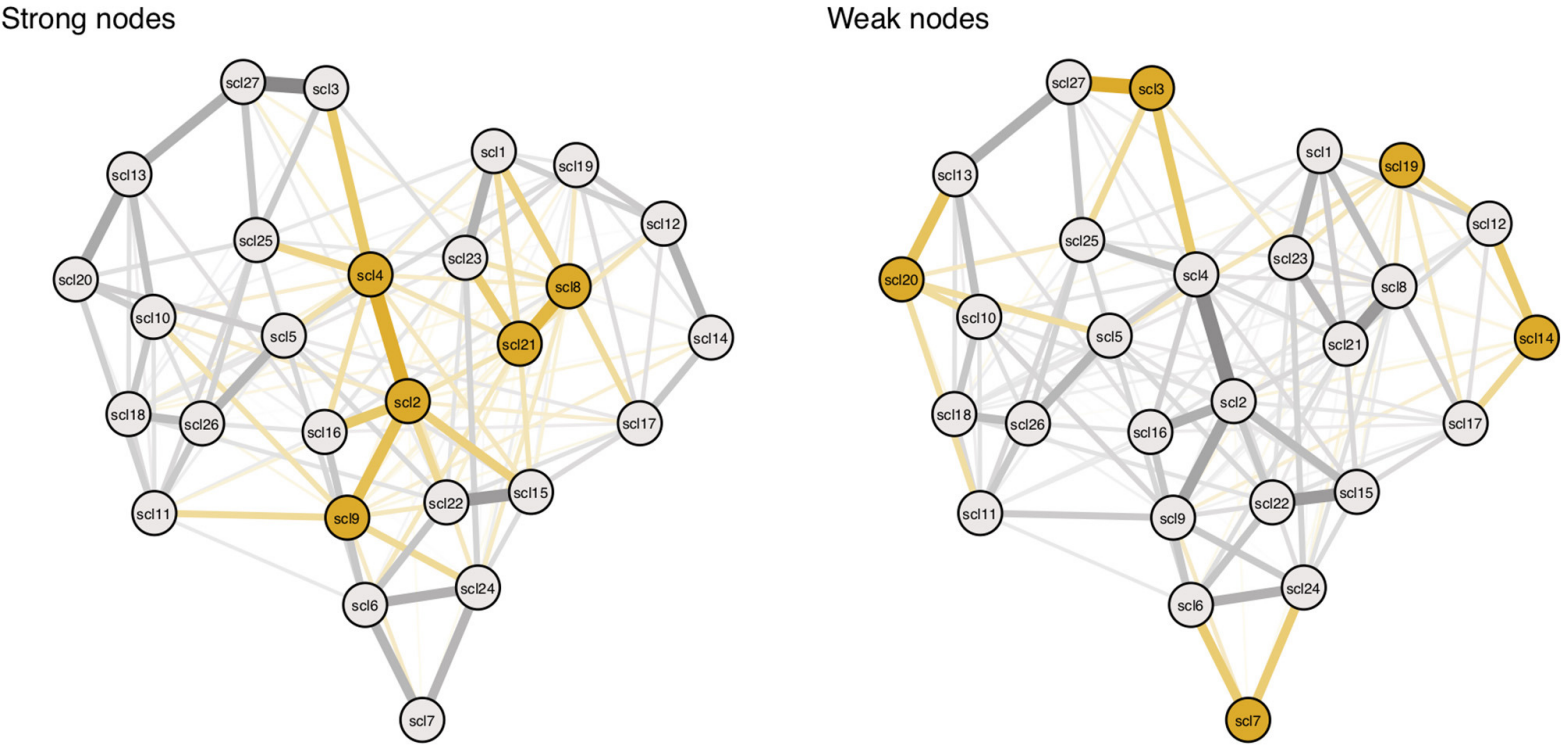
Targeting parameters of strong and weak nodes. Yellow nodes and edges represent targets in the simulation. Targets are based on nodes with highest (left panel) and lowest (right panel) node strength. Symptom descriptions can be found in Table 3 in Appendix 1 in the Supplementary Material.

We create two conditions, the strong node condition, and weak node condition. In both conditions, threshold and connectivity parameters are systematically altered using the same 11 multiplying constants from Study I. In the strong nodes condition, parameters belonging to strong nodes are altered, and in the weak nodes condition, parameters belonging to weak nodes are altered (see Figure 8; yellow edges and nodes represent connectivity parameters and threshold parameters that are altered for every condition). For every alteration, symptom activation is simulated using the *IsingSampler* package (46), and ESA and SAS are calculated from these simulated symptom dynamics.

### Results

**Figure 9:**
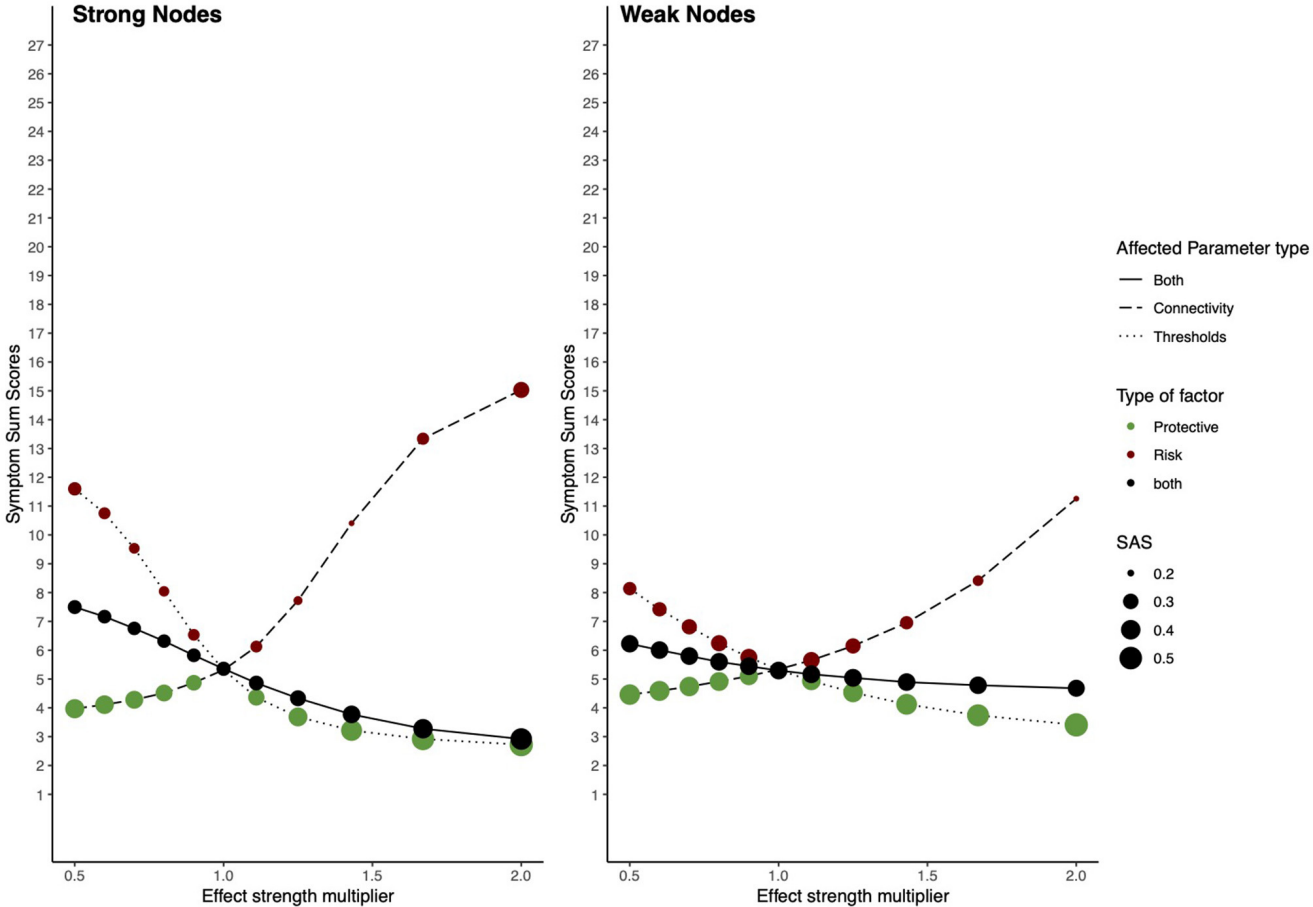
Risk and protective factors affecting parameters of strong and weak nodes. The behavior of an estimated Ising model under influences of hypothetical RP factors, when parameters belonging to nodes with high and weak node strength are targeted. The left panel shows the network behavior in the strong node condition; the right panel shows its behavior in the weak node condition. The x-axis denotes the value of the multiplier constant. The y-axis denotes ESA. Line type represents which parameters are multiplied; threshold parameters, connectivity parameters, or both. The color of circles represents the hypothetical RP factor that influences network parameters: red circles represent risk factors, green circles, protective factors, and black circles represent both factors. The size of the circles represents SAS.

Here we will discuss the general results from the simulation study. Figure 9 shows the complete results, including all alterations on the network architectures, and Table 2 shows the results for the extreme values influences from RP factors, i.e., when the constant used as multiplier is equal to 0.5 or 2.

**Table 2:** Risk and protective factors influencing strong or weak nodes.

**Factor type**	**Strong nodes**		**Weak nodes**		**Multiplier**
	***ESA***	***SAS***	***ESA***	***SAS***	
Baseline	5.32	0.27	5.34	0.27	1
Moderator					
Risk	15.03	0.31	11.26	0.18	2
Protective	3.97	0.39	4.46	0.32	.5
Main Effect					
Risk	11.60	0.26	8.14	0.22	.5
Protective	2.72	0.53	3.41	0.38	2
Both	7.50	0.27	6.22	0.27	.5
	2.92	0.46	4.68	0.26	2

*ESA stands for Expected Symptom Activity, which describes the symptom activity levels. ESA ranges between 0 and number of symptoms, in this case, 27. Low ESA indicates low activity levels, meaning the system is in a healthy state. SAS stands for Symptom Activity Stability, which describes the stability of symptom activity levels. SAS is computed by taking the inverse standard deviation. An SAS of 1 indicates a standard deviation of 1, SAS <1 indicates decreasing stability (increasing standard deviation), and SAS >1 indicates increasing stability (decreasing standard deviation). Low SAS indicates unstable symptom activity patterns. A system is resilient when ESA is low and SAS is high, meaning that the system has a stable and low level of symptom activity*.

Baseline ESA (i.e., when the constant used as multiplier = 1) for the model is low, meaning that the sample is healthy. However, baseline SAS is also low, meaning that this healthy state is unstable. In the strong nodes condition, risk factors strongly increase ESA and maintain SAS, meaning that they push the network toward a state of higher symptom activity, however, maintaining its instability. Protective factors decrease ESA and increase SAS, meaning they push the system toward a resilient state. When RP factors target connectivity and threshold parameters simultaneously, dynamics fluctuate within a wider range of ESA and SAS, nonetheless, maintaining a relative healthy and stable state.

In the weak nodes condition, RP factors have a smaller effect on resilience. Risk factors increase ESA; however, they have a weaker effect compared to the strong nodes condition. SAS is further decreased, meaning that the system is pushed toward an unstable state of moderate symptom activity. Protective factors decrease ESA but have a more moderate effect on lowering ESA compared to the strong nodes condition. When both RP factors target connectivity parameters and threshold parameters simultaneously, they maintain SAS on its baseline level, while ESA fluctuates within a smaller range compared to the strong nodes condition.

### Discussion

We conclude that it matters which parameters are targeted by RP factors. RP factors altering parameters belonging to strong nodes have a more substantial effect on resilience than weak nodes. The range of ESA is wider in the strong nodes condition than in the weak nodes condition. Our study shows that risk factors in the strong nodes condition have a larger effect on ESA and SAS than risk parameters in the weak nodes group. However, this group difference does not hold for protective factors. This could be related to the health of the used sample, where baseline ESA is low.

Specific relations between RP factors and symptoms need to be estimated on the individual symptom level to understand how RP factors affect resilience. Therefore, in the next study, the effect of RP factors on resilience will be calculated by estimating a model from empirical data on RP factors and symptoms. This means that the associations between RP factors and specific symptoms will be empirically estimated. In this way, the effect of RP factors on specific symptoms can be studied.

## Study III: Empirical Illustration of a System Including Symptoms and RP Factors

In this section, we present an empirical illustration of how the proposed system can be implemented in a model that is estimated from data, including measurements on RP factors and psychiatric symptoms. We investigate which specific RP factors are associated with specific symptoms, and how symptom activity levels change when they are targeted by associated RP factors.

Contrary to the former two simulation studies, no data will be generated, nor will network architecture be altered on hypothetical target points. Instead, we estimate a network that includes both symptoms and the RP factors which allows us to study possible symptom-specific pathways with RP factors and the system as a whole.

### Study Design

#### Data

We use the same dataset as the former study and include the measurements on RP factors from the same participants. RP factors are determined a priori; meaning factors are labeled as “risk” or “protective” before data are collected. Risk factors include measurements on tobacco use, alcohol use, and illicit drug use. Protective factors include measurements on physical activity, religious practice, sexual life satisfaction, and volunteer work.

Variables in this dataset are measured on different scales. The variables physical activity, tobacco use, alcohol use, and illicit drug use are measured on a binary scale, religious practice and volunteer work are measured on an ordinal scale (five levels), and sexual life satisfaction is measured on an ordinal scale (six levels). All variables are recoded such that “0” indicates no presence of the variable and “1” or higher indicates (increasing) presence. SCL-27 items representing symptoms (56) are measured on an ordinal scale (five levels).

Due to high correlations between the three risk factors, tobacco use, alcohol use, and illicit drug use, these factors have been collapsed into one risk factor, “substance use.” This was done by summing over all three factors, which originally were measured on a binary scale, where 0 indicated no usage and 1 indicated usage. The novel “substance use” variable ranges from 0 to 3.

#### Model

In order to account for the different measurement scales used in the data, a Mixed Graphical Model (MGM; 62) is estimated. This network model includes both categorical and continuous variables. Here we choose to model ordinal variables as continuous variables.

The model uses nodewise regression to calculate associations between nodes (63). For every variable, its intercept, and the beta-coefficients of all other variables are computed. This intercept represents the threshold of the node, and the beta-coefficients represent connectivity parameters with neighboring nodes. Regularization is applied to select the sparsest model, meaning that most edges with small values are pushed toward zero to control for false-positive edges (37).

The MGM estimates which variables are positively or negatively associated with each other. These associations represent main effects: if, for example, the variables “alcohol use” and “SCL-2: feeling blue” are positively connected, this means that if “alcohol use” increases, “SCL-2: feeling blue” increases as well. Keep in mind that this relationship could also be the other way around, which we will discuss further in the section General Discussion. The MGM is estimated using the *bootnet* package in R with the *mgm* default, using 10-fold cross-validation to select the regularization parameter (61).

Moderation analysis is used to study which RP factors could influence connectivity parameters of the symptom network. This analysis checks for every relationship between RP factors and symptoms if another variable moderates this relationship. This is done by estimating a *Moderated Network Model* [MNM; (64)], using the *mgm* package in R (63).

#### Assessing Resilience

In this study, we investigate how symptom activity levels change due to the presence or absence of RP factors. To study how RP factors affect symptom activity levels and stability, we condition on different values of these RP factors. Lowest values of RP factors indicate absence, highest values indicate their presence. The means of symptoms and possibly also the interactions between symptoms can be functions of the RP factors. If we condition on the RP factors we fix them to specific values, which affects the means and possibly interactions between symptoms. The effect of RP factors' presence or absence is calculated by conditioning on these RP factor values^2^. For example, conditioning on the presence of the protective factor “volunteer work,” is done by conditioning on its highest value, which is 5. The rest of the RP factors maintain their mean value. Based on the model, the novel symptom means are computed for the situation where “volunteer work” has a value of 5.

We investigate two situations by conditioning on the RP factors. In the first situation, we condition on the presence of protective factors, meaning item scores on protective factors are ≥ 1, and absence of risk factors, meaning item scores on risk factors are 0. Second, we study the opposite situation, namely, the presence of risk factors and the absence of protective factors. In both situations, novel symptom means for all SCL-27 items are computed. Note that it is not necessary that all symptoms will change in their means, since mean changes depend on whether a symptom mean is a function of the RP factors. In other words, if a symptom such as “SCL-6: your mind going blank” is not associated with any RP factors, and neither are its neighboring symptoms, the SCL-6 symptom mean will not change despite conditioning on any RP factor.

To compare symptom activity levels from the baseline model with the two conditioned situations representing the presence and absence of specific RP factors, ESA is computed in the baseline model and two conditioned models. Baseline symptom activity can be calculated from the data by calculating the individual symptom means of all the SCL-27 items. The novel, conditioned symptom means are computed after conditioning on the presence/absence of the RP factors. ESA is calculated by summing over all (conditioned) symptom means.

SAS will not be computed since, in the current analysis, ESA variance does not relate to symptom activity stability. Conditioning on RP factors does not change the variance patterns in symptoms. To compute SAS, the probability distribution of the whole model needs to be known, which is problematic in its current set-up because data are measured on a larger scale compared to the Ising model's binary case. A possible solution for future research is to gather longitudinal data, as will be further discussed in the section General Discussion.

To interpret current analyses outcomes using results from the former theoretical simulations, network density and node strength centrality of the symptom nodes will be computed. Density will only be computed for edges between symptom nodes.

### Results

**Figure 10:**
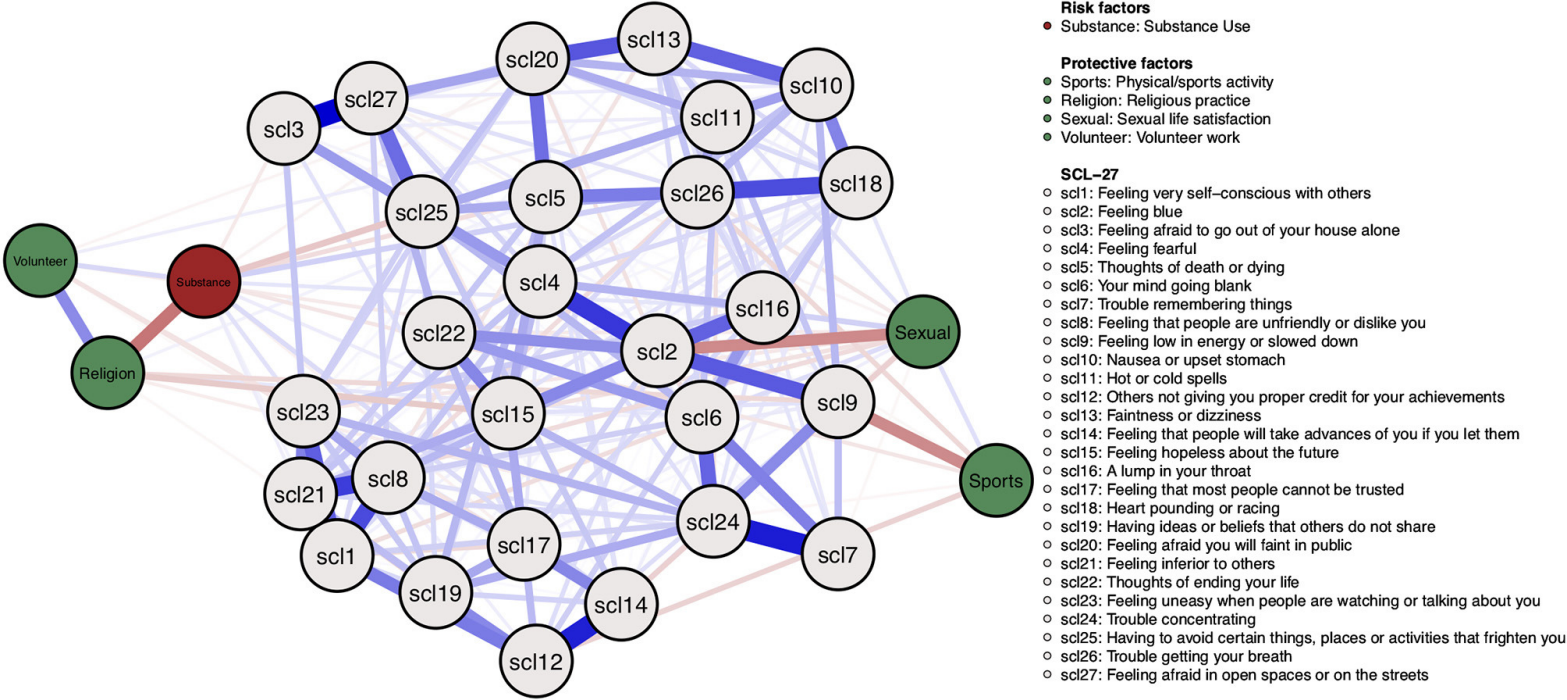
Mixed graphical model including symptoms and risk and protective factors. Gray nodes represent SCL-27 symptoms. Green nodes represent protective factors; red nodes represent risk factors. Blue edges represent positive associations, and red edges represent negative associations. The width of edges and color intensity represents edge strength.

Figure 10 shows the estimated network model. The risk factor “substance use” is negatively associated with the protective factor “religious practice”, and positively associated with “volunteer work”. Surprisingly, there are also some negative edges between the risk factor and symptoms, such as the SCL-5 symptom “thoughts of death or dying.”

Protective factors are mostly positively associated with each other and negatively associated with symptoms. For example, the protective factor “religious practice” is negatively associated with the SCL-15 symptom “Feeling hopeless about the future,” the protective factor “sports/physical activity” is negatively associated with the SCL-9 symptom “Feeling low in energy or slowed down,” and the protective factor “sexual life satisfaction” is negatively associated with the SCL-2 symptom “feeling blue.”

No moderators between symptoms and RP factors have been found.

The network density of edges between symptom nodes is 0.55. Nodes with the highest node strength are SCL-2, SCL-24, SCL-4, SCL-1, SCL-21. See Appendix 2 of the Supplementary Material for the centrality plot. All strongest symptoms are connected to at least two RP factors, although they have small edges.

The ESA of the baseline model is 36.21. Highest possible ESA is 4 ^*^ 27 = 108. When conditioning on the presence of protective factors and the absence of the risk factor, ESA decreases to 35.45. The difference with baseline ESA is – 0.75. When conditioning on the presence of the risk factor and the absence of protective factors, ESA is 36.70, meaning an increase of 0.49 compared with baseline ESA.

### Discussion

We studied symptom-specific associations with RP factors, and the effect of the presence or absence of RP factors on symptom levels. Overall, protective factors were positively associated amongst each other and negatively associated with specific symptoms. The risk factor “substance use” was mostly positively associated with specific symptoms, however, there were also some negative associations with specific symptoms. No moderators were found.

When conditioning on the presence of the risk factor and the absence of protective factors, ESA slightly increased. Contrary, when conditioning on the presence of protective factors and the absence of the risk factor, ESA slightly decreased. This means that there is a small effect from the RP factors on symptom activity levels, where risk factors slightly decrease and protective factors slightly increase symptom activity.

Note, however, that estimated edges are bidirectional. To investigate causal effects, longitudinal data are needed to estimate a dynamic model. Longitudinal data are furthermore needed to calculate SAS.

A possible explanation for the small effect from the RP factors on ESA is that floor effects might be present since baseline symptom activity levels are low. The sample contains many healthy participants, meaning not enough participants are present showing high symptom activity and strong effects with risk factors. Including clinical patients in the sample might show a wider variety of response patterns and stronger effects when conditioning on RP factors. Furthermore, the symptom network consisted of 27 items, while the RP factors consisted of merely five variables. Important RP factors which have a strong influence on the symptom network might be missing. Future studies could repeat the proposed analyses on a dataset with more RP factors to investigate if stronger effects are found.

The symptom network density is 0.5, meaning the range in which ESA could change is smaller, and the strongest nodes are connected to at least two, but not all, RP factors. Estimated edges have a much smaller value than the theoretical simulations' multipliers, explaining why this empirical illustration shows almost no effect.

## General Discussion

In this paper, we presented a formal system where RP factors from biopsychosocial domains influence resilience by altering the architecture of psychopathology symptom networks. Furthermore, we presented two novel metrics to analyze the resilience of symptom networks. Here, we will discuss these contributions and their clinical implications, together with their limitations, and provide concrete suggestions for future research.

Our presented system builds on the theory by Kalisch et al. (34), who propose that resilience factors could affect the architecture of symptom networks. By doing so, resilience factors change the network's symptom activity patterns and resilience. In this paper, we extended that idea to include both risk and protective factors and took a step forward into formalizing the system. We translated possible ways in which RP factors can affect resilience to specific target points on the symptom network parameters, where we made a distinction between main effects targeting threshold parameters and moderators targeting connectivity parameters. Targets from RP factors are operationalized by multiplying these threshold and connectivity parameters with certain constants, which, based on their magnitude, act as risk or protective factors, thereby deteriorating or improving resilience. As a first formalization, we implemented the system using the Ising model as a statistical model representing the symptom network (36). Furthermore, we provided an empirical illustration of how the system could be implemented in a Mixed Graphical Model (63), which analyzes both categorical and continuous data.

A second contribution of the current paper is that we presented two novel metrics for assessing the resilience of symptom networks: Expected Symptom Activity (ESA) and Symptom Activity Stability (SAS). Computing ESA is based on the common practice in the resilience literature to relate the presence and/or absence of RP factors to symptom severity levels (50, 51). Furthermore, it is consistent with the psychological network literature to compute the number of active symptoms as an indicator of the state of the symptom network (32, 38). However, symptom levels do not indicate how resilient a system is, as a resilient system should maintain or quickly bounce back to its healthy state despite facing adversity (21, 22). Thus, resilience entails a low level of symptom activity and robust stability of this low level. Stability measures have been developed in the field of ecology [e.g., see (66, 67)] and physics [e.g., calculating the Gibbs entropy; (52)] and are crucial for studying the resilience of dynamical systems such as ecosystems. In this paper, we linked concepts from stability theory with existing measures in the resilience literature and psychopathology network theory by proposing to compute the variance of symptom activity patterns as a metric for the *stability* of symptom levels.

Symptom network models including RP factors can have important clinical implications for the analysis of symptom-specific pathways. Symptom network models focus on unique associations between symptoms, which may suggest pathways for which symptom-level intervention strategies can be developed (68). This is especially important for multifactorial disorders such as depression, since scales or sub-scales of these disorders are unstable over time (e.g., they are not measurement invariant), and do not measure one, underlying component (i.e., they are not unidimensional) (60). Therefore, symptom network analysis offers a promising, novel technique to compare the symptom-specific efficacy of treatment interventions for depression, such as antidepressant medication vs. Cognitive Behavioral Therapy [CBT; (69)]. Including RP factors into symptom networks could yield new insights into symptom-specific pathways involving biopsychosocial factors, which aid the development of novel and more effective intervention strategies.

Apart from analyzing symptom-specific pathways in experimental data, a recent call for “precision psychiatry” urges the development of computational models that integrate data units across scales, such as biomarkers, self-report symptom inventories, and clinicians' observations (70–72). The collection of experimental data is costly, which is why an exploratory analysis with observational data gives a first indication of possible symptom-specific pathways between specific symptoms and RP factors, such as biomarkers. In this paper, we showed a simulation-based, exploratory method for observational data, which aims to investigate which symptom-specific pathways might exist with relevant RP factors.

The presented method has some limitations, of which we will discuss the most pressing ones. Using the Ising model as a statistical model to incorporate the theory by Kalisch et al. (34) has limitations, as the model does not hold for more complex elements of the proposed theory. The first and major one is that the dynamical aspect of the theory by Kalisch et al. (34) cannot be investigated with the Ising model. The theory states that the presence of a protective factor could, over time, increasingly increment a symptom's threshold, as the protective factor and symptom get entangled in a positive feedback loop. For example, having a job with regular working hours might lead to better sleep and a smaller chance (i.e., stronger negative threshold) to develop the psychiatric symptom insomnia. As sleep improves, one's job performance might also improve, creating a positive feedback loop between the protective factor (stable job) and stronger symptom threshold (insomnia). To investigate this dynamical aspect, an invariant model such as the Ising model is not suitable.

A second limitation is that the Ising model does not consider different time scales on which the various variables operate. It is plausible to assume that a protective factor such as social support evolves on a slower time scale than a psychiatric symptom such as depressed mood. Future research could expand the proposed system in line with the Personality-Resilience-Psychopathology model by Lunansky et al. (73), where personality variables that operate on a slower time scales affect specific network parameters of fast-evolving symptom networks. A third limitation is that the Ising model can only analyze binary data, while measures on symptoms and RP factors will usually be on an ordinal or continuous scale. To address this limitation, our study also provided an empirical illustration of the proposed system using an MGM (63). However, this is not an optimal solution since the MGM also does not account for the dynamical aspect of the theory. Lastly, a limitation of using the Ising model is that specific aspects of its dynamics are restricted within its domain (53). Some results from our simulation studies are, therefore, only valid within this specific domain. For example, when using a different binary notation for the state of the variables (the {−1,1} domain instead of the {0,1} domain), increasing the density of the network does not increase symptom activation but only its variance. Dynamics of the {0,1} domain or {−1,1} domain can be translated to each other by transforming the network's parameters as described by Haslbeck et al. (53).

We have several concrete suggestions for future research. First, the further development of time-varying models to study holistic models of resilience. Time-varying models allow for dynamic relations between variables over time (74). Differential equations describe how variables change as a function of themselves and other related variables, which is why computational models often use these equations to simulate behavioral patterns over time. For example, the computational model for Panic Disorder by Robinaugh et al. (75) explains how panic attacks can instantiate, reach their peak, and end, by using a mathematical model of differential equations. These equations represent dynamic relationships between relevant variables, such as arousal and perceived threat, and are constructed based on reported relationships in the literature. Second, using latent change models such as the Random Intercept Cross-Lagged Panel Model (76). This model estimates dynamic relations between different variables over time, and could be used to model the effects from RP factors from various domains on psychiatric symptoms. Therefore, future research should focus on collecting longitudinal data, including measures on psychiatric symptoms and various RP factors, and developing and estimating time-varying models.

Second, there are multiple ways in which the proposed metrics, especially SAS, could be improved. As general and straightforward as computing the variance is, it is also not the most exact way of predicting how a system will react in the face of adversity. Furthermore, high variability of a system's behavioral patterns might also be an indicator of strong adaptability (77). Therefore, computing SAS as a resilience indicator could be further extended by computing a symptom network's sensitivity to *perturbations* (78). This would give a more dynamic indicator of the stability and adaptability of symptom activity patterns when faced with perturbations. Alternatively, when developing a more advanced model for the proposed system in this paper using differential equations, the system's potential landscape can be computed [e.g., (79)], giving an exact overview of the system's stable states. This paper outlined the main reasons for computing ESA and SAS as resilience metrics of symptom networks, while their optimal computation will hopefully be further developed in future research.

Holistic, ecosystem models, including variables from multiple domains such as biopsychosocial models, are an interesting candidate for studying the complex nature of mental health and its relationship with various risk and protective factors. By combining ideas and models from the network perspective of psychopathology (32, 36, 38, 63) with the theory on resilience factors targeting network parameters (34), we took one step forward toward the formalization of these models.

## Data Availability Statement

Publicly available datasets were analyzed in this study. These data, including the R-scripts for their analyses, can be found here: https://osf.io/jhzk3/.

## Ethics Statement

Ethical review and approval was not required for the study on human participants in accordance with the local legislation and institutional requirements. The patients/participants provided their written informed consent to participate in this study.

## Author Contributions

GL designed the study. GL, CB, JH, ML, and DB wrote the manuscript. GL, CB, and JH analyzed the data. CG and ME collected the data. DB supervised the project. All authors discussed the results and contributed to the final manuscript.

## Conflict of Interest

The authors declare that the research was conducted in the absence of any commercial or financial relationships that could be construed as a potential conflict of interest.

## Footnotes

^1^Note that SAS can be low when ESA is low or when ESA is high. Although the symptom network is stable in both cases, it is not resilient in its healthy state for the latter case. Also note in many cases there will be a strong relation between ESA and SAS, in the sense that SAS will be lowest if ESA hovers around *n*/2 and will increase as ESA approaches its limits at 0 or *n*. However, one can also set up parameter settings for the network in which ESA equals *n*/2 and SAS is high (this will occur, for instance, if half of the symptoms has a very strong threshold and the other has a very weak threshold); hence, even though ESA and SAS will often be related, this is not necessarily so.

^2^Mathematically, in the regression formula used for calculating symptom means, the beta-coefficient (representing the connectivity parameter) of the corresponding RP factor is multiplied by a specific value of this RP factor instead of on the RP factor's mean. The effect of RP factors' presence or absence is calculated by conditioning on these RP factor values. However, when estimating the MGM, variables are standardized. This means model parameter estimates need to be transformed in order to be unbiased for computing ESA on the data scale (65). Take β to be the unbiased beta-coefficients where: β = (β_1_, β_2_ , . . . β_*p*_) and $\hat{\beta }$the biased beta-coefficients. The unbiased beta-coefficients can be calculated with: β = $\hat{\beta }{\;}^{*}$ $\frac{\sigma y}{\sigma x}$, where σ*y* and σ*x* are the original data standard deviations. The unbiased intercept can be calculated with: ${\hat{\beta }}_{0}=\;\bar{y}-\;\overset{p}{\underset{j=1}{\sum }}{\bar{x}}_{j}{\hat{\beta }}_{j}$, where $\bar{y}$ and ${\left\{{\bar{x}}_{j}\right\}}_{1}^{p}$ are the original data means.
