# Supplementary material for: The Mental Health Ecosystem: Extending Symptom Networks With Risk and Protective Factors
Source: Front Psychiatry. 2021 Mar 18;12:640658. doi: 10.3389/fpsyt.2021.640658 (PMC8012560; doi:10.3389/fpsyt.2021.640658)
Supplement: Supplementary file 1 [file Data_Sheet_1.docx]

**Supplementary materials**

**Appendix 1**

**Table 3**

*SCL-27 Variable Names*

| Variable label | Variable description |
| --- | --- |
| SCL-1 | Feeling very self-conscious with others |
| SCL-2 | Feeling blue |
| SCL-3 | Feeling afraid to go out of your house alone |
| SCL-4 | Feeling fearful |
| SCL-5 | Thoughts of death or dying |
| SCL-6 | Your mind going blank |
| SCL-7 | Trouble remembering things |
| SCL-8 | Feeling that people are unfriendly or dislike you |
| SCL-9 | Feeling low in energy or slowed down |
| SCL-10 | Nausea or upset stomach |
| SCL-11 | Hot or cold spells |
| SCL-12 | Others not giving you proper credit for your achievements |
| SCL-13 | Faintness or dizziness |
| SCL-14 | Feeling that people will take advances of you if you let them |
| SCL-15 | Feeling hopeless about the future |
| SCL-16 | A lump in your throat |
| SCL-17 | Feeling that most people cannot be trusted |
| SCL-18 | Heart pounding or racing |
| SCL-19 | Having ideas or beliefs that others do not share |
| SCL-20 | Feeling afraid you will faint in public |
| SCL-21 | Feeling inferior to others |
| SCL-22 | Thoughts of ending your life |
| SCL-23 | Feeling uneasy when people are watching or talking about you |
| SCL-24 | Trouble concentrating |
| SCL-25 | Having to avoid certain things, places or activities that frighten you |
| SCL-26 | Trouble getting your breath |
| SCL-27 | Feeling afraid in open spaces or on the streets |

**Appendix 2**

*Centrality plot showing node strength of SCL-27 nodes*

*Note.* The x-axis shows node strength on standardized z-scores, the y-axis shows all SCL-27 variables. The upper variables have highest node strength, and the lower variables have lowest node strength.
